# Germinated soy germ with increased soyasaponin Ab improves BMP-2-induced bone formation and protects against *in vivo* bone loss in osteoporosis

**DOI:** 10.1038/s41598-018-31118-w

**Published:** 2018-08-28

**Authors:** Chan-Woong Choi, Sik-Won Choi, Han-Jun Kim, Kwang-Sik Lee, Shin-Hye Kim, Sun-Lim Kim, Sun Hee Do, Woo-Duck Seo

**Affiliations:** 1 College of Veterinary Medicine, Konkuk University, Seoul, 05029 Republic of Korea; 2 National Institute of Crop Science, Rural Development Administration, Wanju-Gun, 55365 Republic of Korea; 3 College of Crop Science and Biotechnology, Dankook University, Cheonan, 31116 Republic of Korea; 4 Department of Biological Sciences, College of Natural Science, Chonbuk National University, Jeonbuk, 55000 Republic of Korea; 5 Department of Central Area Crop Science, National Institute of Crop Science, Rural Development Administration, Suwon, 16429 Republic of Korea

## Abstract

Osteoporosis is frequently induced following menopause, and bone fractures result in serious problems including skeletal deformity, pain, and increased mortality. Therefore, safe and effective therapeutic agents are needed for osteoporosis. This study aimed to clarify the bone protecting effects of germinated soy germ extracts (GSGE) and their mode of action. GSGE increased expression of alkaline phosphatase (ALP) and osteocalcin (OCL) by stimulating the expression of runt-related transcription factor 2 (Runx2) and osterix (Osx) through activation of Smad signaling molecules. Furthermore, germination of soy germ increased levels of nutritional components, especially soyasaponin Ab. The anabolic activity of soyasaponin Ab in GSGE was also evaluated. GSGE and soyasaponin Ab significantly protected against ovariectomy (OVX)-induced bone loss and improved bone-specific alkaline phosphatase (BALP) level in mouse serum. These *in vitro* and *in vivo* study results demonstrated that GSGE and soyasaponin Ab have potential as therapeutic candidate agents for bone protection in postmenopausal osteoporosis.

## Introduction

Menopause is the permanent cessation of menstruation when reproductive capacity ceases. The ovaries stop functioning, and production of steroid and peptide hormones decreases. This process influences hormonal changes in many organ systems^1^. Osteoporosis is a common disease associated with postmenopausal syndrome and can have a chronic effect on the health of middle-aged women. Osteoporosis or osteopenia is reported to occur in half of menopausal women^2^. Osteoporosis develops from an imbalance between bone formation and bone resorption that results in low bone mass and microarchitectural deterioration of bone, leading to increased susceptibility to bone fractures. In general, hormone replacement therapy has been used to prevent and treat osteoporosis in postmenopausal women. Hormone replacement therapy is reported to decrease the risk of osteoporosis by about 50% at the onset of menopause and to have long lasting effects^3^. However, studies report that hormone replacement therapy could increase the potential risk of adverse effects such as breast tenderness, thromboembolic disease, stroke, cardiovascular disease, breast cancer, and endometrial cancer^4^.

Studies are actively being conducted to improve symptoms of menopausal women in order to minimize adverse effects caused by hormone replacement therapy. Among the candidate treatments, soy is receiving attention as a complementary and alternative medicine that might be used for long-term treatment of menopausal disease. Studies have been conducted on the potential efficacy and various effects of soy and soy germ such as on climacteric symptoms, antioxidant capacity, spatial memory performance, and inflammatory bowel disease^5–8^. However, most previous research studied isoflavones from soy or soybeans and their effects on postmenopausal symptoms. Reports indicate that isoflavones act as phytoestrogens due to their structural similarity to estrogen.

The germination process can influence the type of biologically active substances, levels of nutritional components, and palatability of soybeans^9^. In spite of biochemical and nutritional changes during germination, few efficacy studies have been conducted on germinated soy germ. To the best of our knowledge, no efficacy study has been performed on the molecular mechanism of postmenopausal osteoporosis using germinated soy germ and its functional components. Therefore, the aim of this study was to assess the effects of germinated soy germ extracts (GSGE) and its pharmaceutical components on osteoblast differentiation and postmenopausal osteoporosis using an ovariectomized animal model.

## Results

### GSGE stimulates BMP-2-induced osteoblast differentiation in C2C12 cells

**Figure 1:**
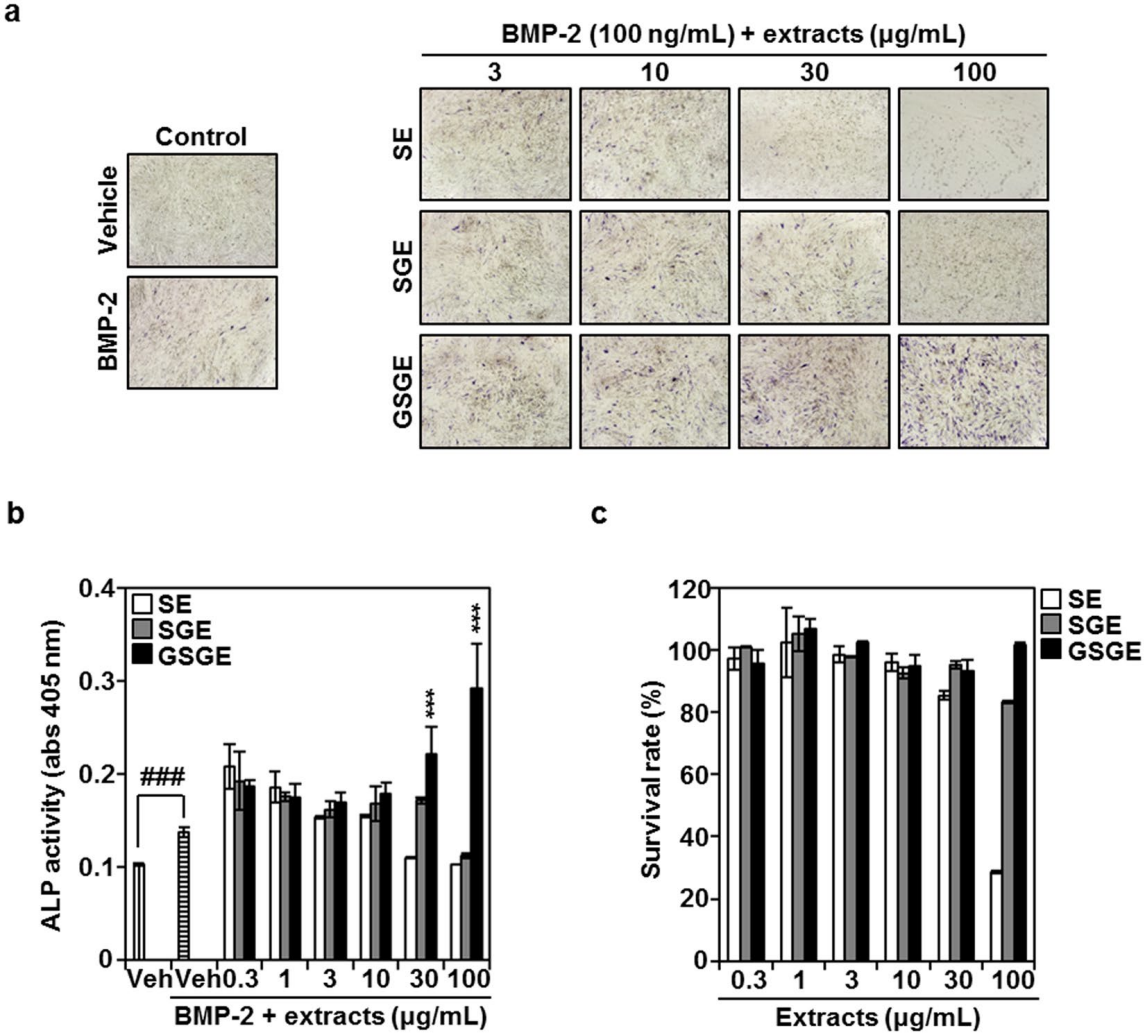
GSGE enhances osteoblast differentiation. (**a**) C2C12 cells were cultured for 3 days with BMP-2 (100 ng/mL) with either vehicle (prethanol A) or the indicated concentration of SE, SGE, or GSGE. Osteoblast differentiation was visualized by ALP staining. (**b**) ALP activity was measured at 405 nm absorbance. ^###^*p* < 0.001 (versus control); ***p < 0.001 (versus BMP-2-treated group). (**c**) Effects of SE, SGE, and GSGE on C2C12 cell viability were evaluated by CCK-8 assay. Data are mean ± SD and are representative of at least three experiments.

To study the effects of germinated soy germ on BMP-2-mediated osteogenesis, C2C12 cells were incubated with various concentrations of soy, soy germ, or germinated soy germ extract, followed by BMP-2 (100 ng/mL). As shown in Fig. 1a, GSGE induced ALP expression in a dose-dependent manner in the presence of BMP-2, but enhancement of SE and SGE did not have an effect. Consistent with this result, GSGE significantly enhanced BMP-2-stimulated ALP activity in a dose-dependent manner (Fig. 1b). SGE and GSGE did not show cytotoxicity, but SE inhibited C2C12 cell proliferation at 100 μg/mL (Fig. 1c). These results suggest that GSGE significantly promoted BMP-2-dependent osteoblast differentiation compared with induction by SE and SGE.

### GSGE promotes BMP-2-stimulated expression of transcription factors Runx2 and Osx in osteoblast differentiation

**Figure 2:**
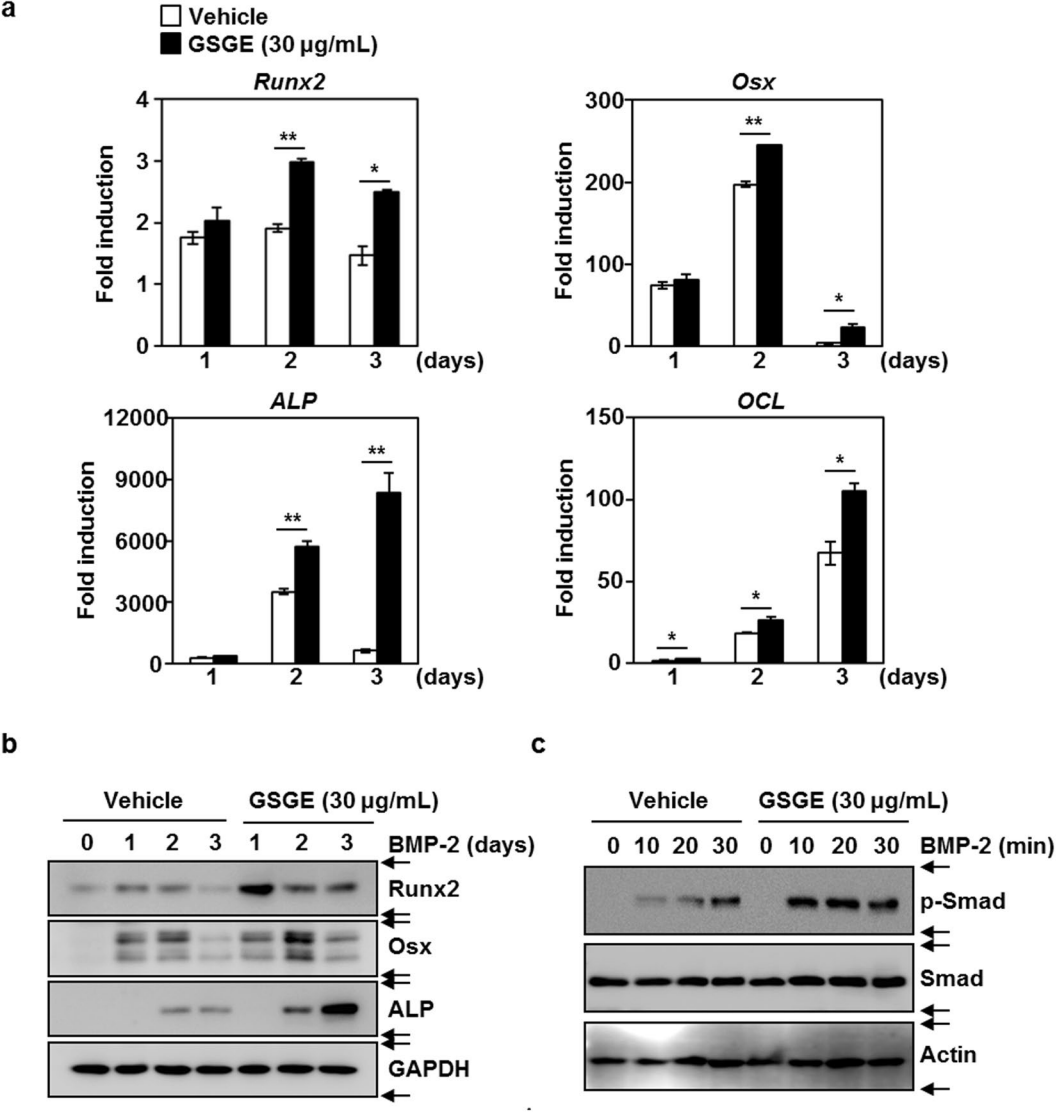
GSGE stimulates BMP-2-induced expression of Runx2 and Osx and accelerates BMP-2-mediated phosphorylation of Smad signalling molecules. (**a**) C2C12 cells were induced with BMP-2 (100 ng/mL) in the presence or absence of GSGE (30 μg/mL) for the indicated times. Total RNA was then isolated using TRIzol reagent, and the mRNA expression levels were evaluated using real-time PCR. GAPDH or HPRT was used as the internal control. *p < 0.05; **p < 0.01 (versus vehicle control). (**b**) Effects of GSGE on BMP-2-stimulated transcription factor proteins were evaluated using Western blot analysis. GAPDH was used as the internal control. Data are representative of at least three experiments. (**c**) In conditions of serum starvation for 1 day, C2C12 cells were pretreated with or without GSGE (30 μg/mL) for 1 hour before BMP-2 stimulation (100 ng/mL) for the indicated time. Expression of Smad molecules was evaluated using Western blot analysis. One representative result from three independent experiments yielding similar results is shown.

The stimulatory effect of GSGE on osteoblast differentiation was examined by evaluating the expression of osteogenesis-related genes for transcription factors. BMP-2 induced mRNA of the osteoblast differentiation-mediated transcription factors Runx2 and Osx. This induction was synergistically enhanced by addition of GSGE (30 μg/mL). Levels of transcription factor-regulated molecules ALP and OCL were also enhanced on the indicated days (Fig. 2a). Western blots showed that BMP-2-induced translation of Runx2, Osx, and ALP was synergistically stimulated by GSGE treatment (Fig. 2b). These results show that the anabolic activity of GSGE had the potential to stimulate the expression of Runx2 and Osx required for osteoblast differentiation.

### GSGE contributes to BMP-2-mediated Smad signaling pathways

To investigate the underlying mechanism of the anabolic effect of GSGE, we examined if GSGE affected induction of a BMP-2-related signaling pathway associated with regulation of Runx2 and Osx expression, which are key transcription factors. The addition of GSGE synergistically enhanced BMP-2-induced phosphorylation of Smad at the indicated times (Fig. 2c).

### Identification and characterization of soyasaponin Ab in GSGE

**Figure 3:**
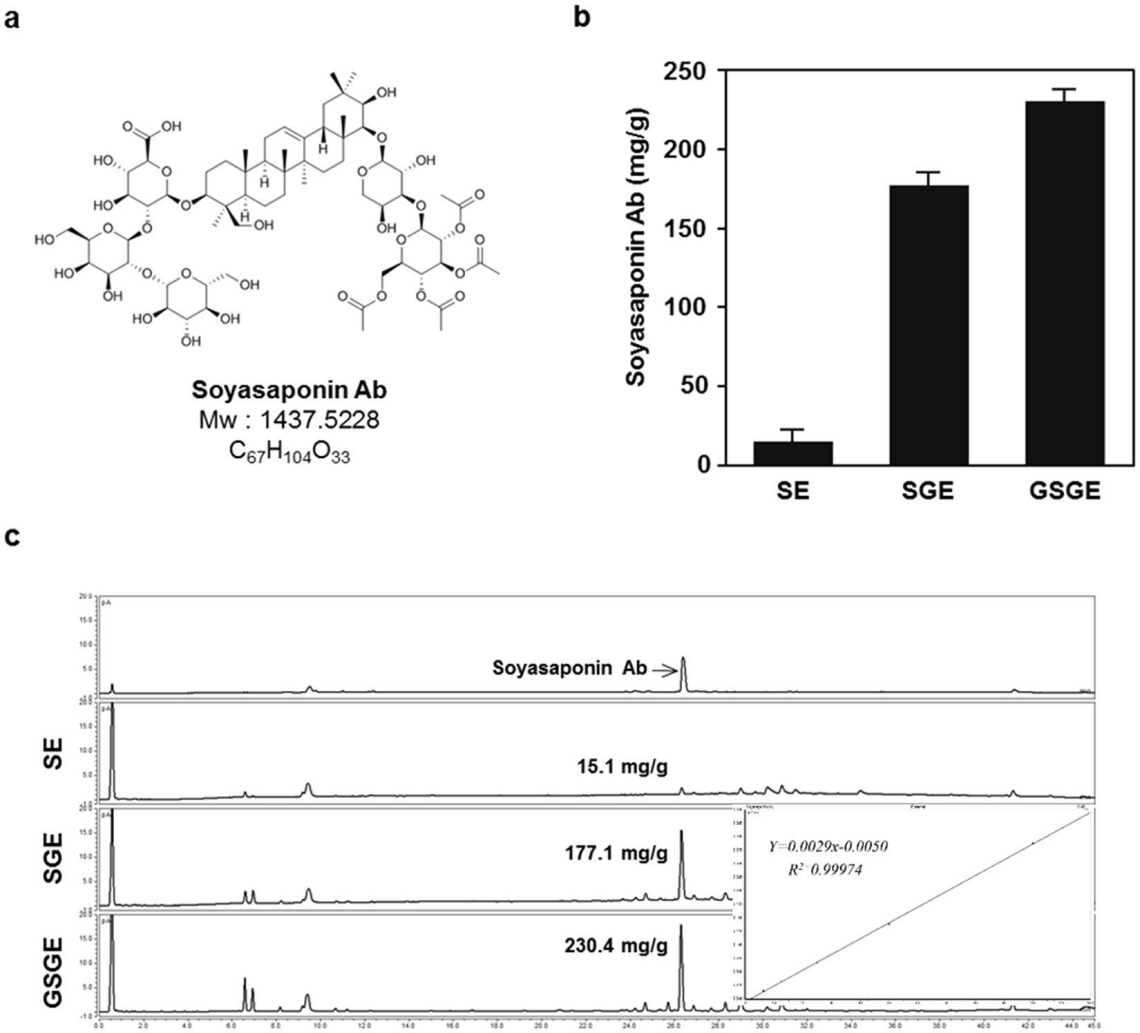
Chemical structure and content analysis of soyasaponin Ab from SE, SGE, and GSGE by UHPLC-CAD. (**a**) Chemical structure and molecular weight of soyasaponin Ab. (**b**) Soyasaponin Ab contents of SE, SGE, and GSGE. (**c**) UHPLC chromatogram of SE, SGE, GSGE and the calibration curve data.

To identify the bioactive compound in GSGE, the composition of major chemicals of GSGE was investigated by UHPLC. The major peak in GSGE was identified as soyasaponin Ab (Fig. 3a) by UPLC-Q-TOF/MS/MS and NMR spectra (Supplementary Fig. S1). Amount of soyasaponin Ab were assessed by UHPLC chromatograms of SE, SGE, and GSGE (Fig. 3c and Supplementary Table S2). As shown in Fig. 3b, the soyasaponin Ab content was 15.1 ± 7.5 mg/g for SE, 177.1 ± 8.2 mg/g for SGE, and 230.4 ± 7.4 mg/g for GSGE. Thus, the soyasaponin Ab content of GSGE was 15 times higher than that of SE and 1.3 times higher than that of SGE. These results indicate that GSGE contains a higher soyasaponin Ab content than SE or SGE.

### Soyasaponin Ab stimulates BMP-2-dependent osteogenesis by Runx2 and Osx activation

**Figure 4:**
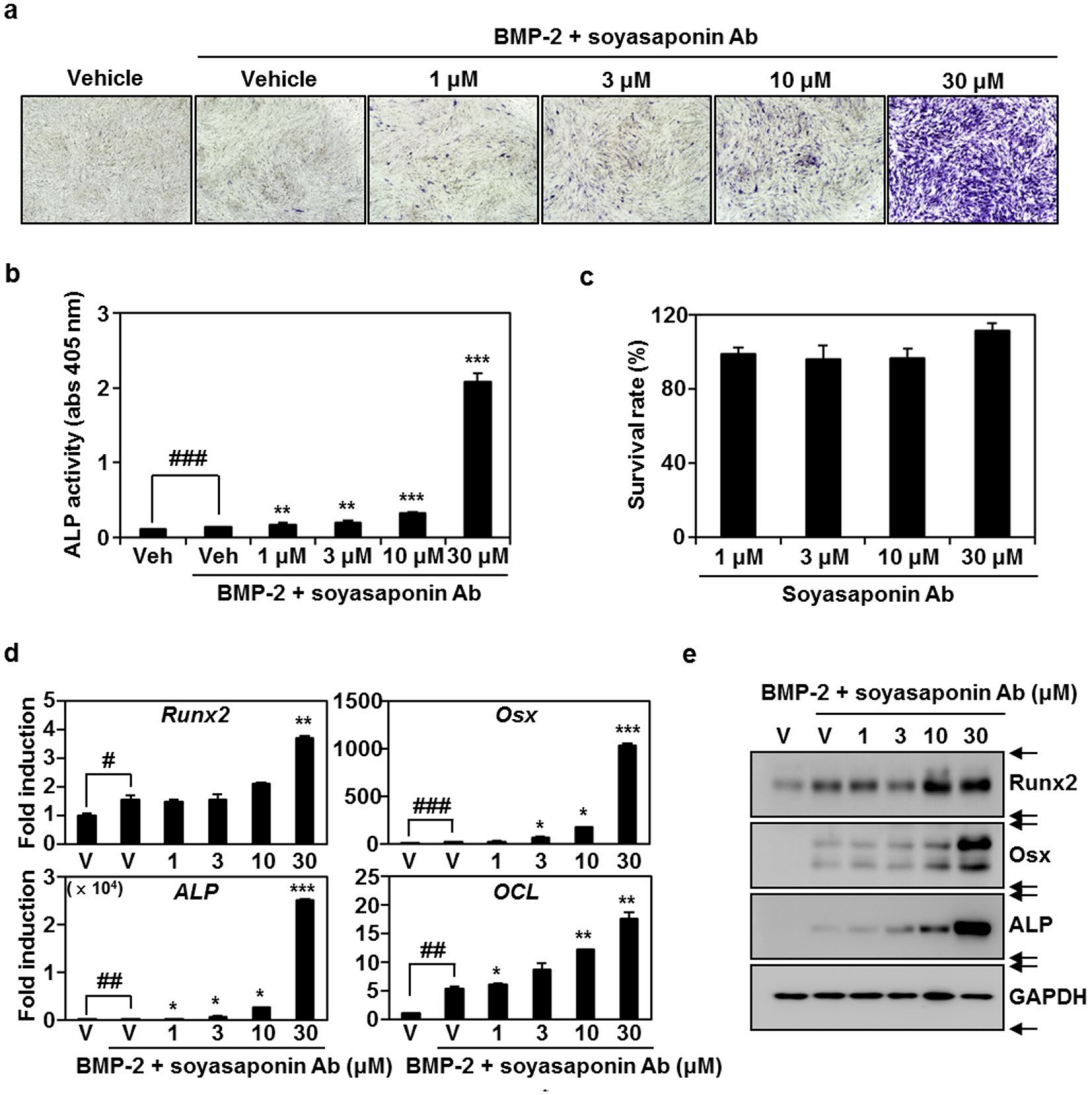
Soyasaponin Ab in GSGE increases osteoblast differentiation by inducing Runx2 and Osx. (**a**) Effect of soyasaponin Ab on BMP-2-induced ALP staining was visualized and evaluated by measuring ALP activity (**b**) ^###^*p* < 0.001 (control); **p < 0.01; ***p < 0.001 (versus BMP-2-treated group). (**c**) Effect of soyasaponin Ab on C2C12 cell viability. (**d**,**e**) Indicated mRNA and protein expression levels were evaluated at the indicated concentrations by real-time PCR or Western blot analysis. Relative fold change in mRNA compared to control is presented. ^#^*p* < 0.05; ^##^*p* < 0.01; ^###^*p* < 0.001 (versus control); *p < 0.05; **p < 0.01; ***p < 0.001 (versus BMP-2-treated group). Data are representative of at least three experiments.

To examine if soyasaponin Ab from GSGE was the active ingredient for increasing osteoblast differentiation, its effect on the BMP-2-mediated commitment of C2C12 cells into osteoblasts was evaluated by expression and activity of ALP and transcription factors for osteogenesis. Soyasaponin Ab significantly stimulated BMP-2-induced ALP expression in a dose-dependent manner (Fig. 4a). Soyasaponin Ab also increased the activity of ALP, a biomarker of osteoblast differentiation, in the presence of BMP-2 (Fig. 4b). Soyasaponin Ab did not affect C2C12 cell survival, indicating that the anabolic effect of soyasaponin Ab was not due to cytotoxic effects (Fig. 4c). To further evaluate the bone-formation effects of soyasaponin Ab, expression of osteoblastogenic molecules was evaluated by real-time PCR and immunoblots after cell treatment with soyasaponin Ab. As shown in Fig. 4d, the treatment with soyasaponin Ab induced the mRNA of Runx2, Osx, ALP, and OCL on differentiation day 3. In addition, treatment with soyasaponin Ab for 3 days dose-dependently stimulated translation of Runx2, Osx, and ALP in the presence of BMP-2 (Fig. 4e). These results demonstrated that isolated soyasaponin Ab from GSGE could be a bioactive ingredient for accelerating osteoblast differentiation.

### GSGE protects ovariectomy-induced bone loss *in vivo*

**Figure 5:**
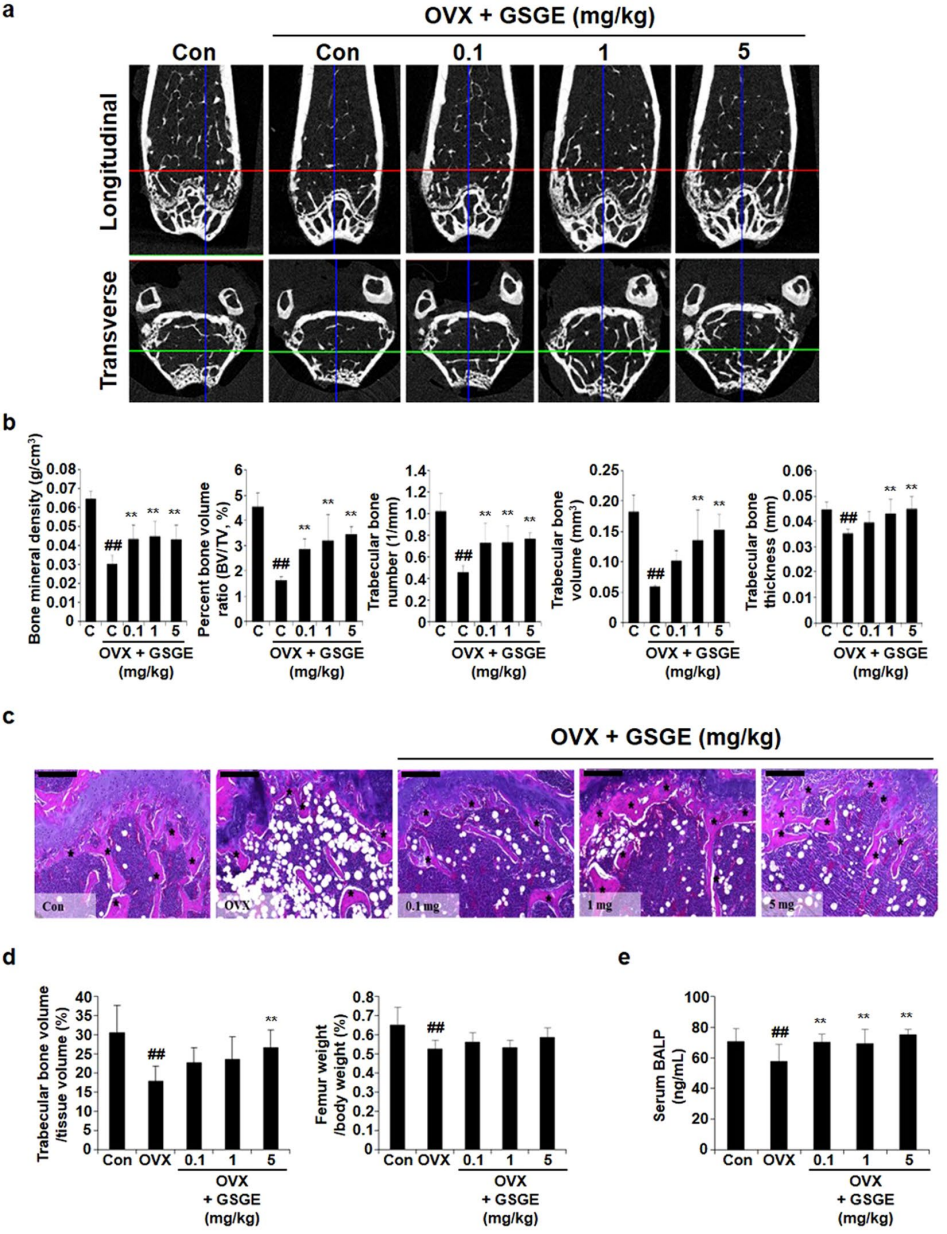
GSGE prevents OVX-induced bone loss *in vivo*. (**a**) Longitudinal and transverse µ-CT images of Con and OVX at 0.1 mg, 1 mg, and 5 mg from left to right, and (**b**) bone mineral density, percent bone volume ratio, trabecular bone number, trabecular bone volume, and trabecular bone thickness of femur measurements by 3D images analyzer. ^##^*p* < 0.01 (versus Con group); **p < 0.01 (versus OVX group). (**c**) Mice were euthanized 12 weeks after GSGE treatment, and femurs were dissected, fixed, decalcified, and sectioned. Sections were stained with H&E. Stained bone sections were photographed under a light microscope (scale bar, 200 μm). (**d**) Femur trabecular volume/tissue volume and femur weight/body weight were analyzed using the Visus image analysis program. ^##^*p* < 0.01 (versus Con group); **p < 0.01 (versus OVX group). (**e**) Blood was collected from abdominal veins through the laparotomy incision. BALP concentration was estimated using an ELISA kit. ^##^*p* < 0.01 (versus Con group); **p < 0.01 (versus OVX group).

The anti-osteoporotic effect of GSGE was evaluated *in vivo* in an osteoporosis model with ovariectomy. As shown in Fig. 5a, μ-CT indicated that trabecular bone in the distal metaphysis of the femur was decreased by OVX, and intragastric injection of GSGE restored OVX-induced trabecular bone loss. OVX-mediated changes in bone mineral density, percent bone volume ratio, trabecular bone number, trabecular bone volume, and trabecular bone thickness were substantially prevented by GSGE (Fig. 5b). Histological assessments revealed that relative trabecular bone dimensions after GSGE treatment were higher than in an OVX group (Fig. 5c). GSGE treatment increased the formation of trabecular bone compared to an OVX group (Fig. 5d; left graph). In addition, femur weight/body weight in the animals who received GSGE treatment were higher than those in the OVX group (Fig. 5d; right graph). To determine the relevance of GSGE for protection against OVX-induced bone loss, the concentration of biomarkers in serum was examined. The level of serum BALP, an indicator of osteoblast activation, was significantly decreased in the OVX group compared to the Con group, but GSGE treatment increased serum BALP level compared to the OVX group (Fig. 5e). Based on these results, we concluded that GSGE has an anabolic effect on an osteogenesis mechanism and bone formation *in vivo*.

### Soyasaponin Ab restores OVX-stimulated osteoporosis

**Figure 6:**
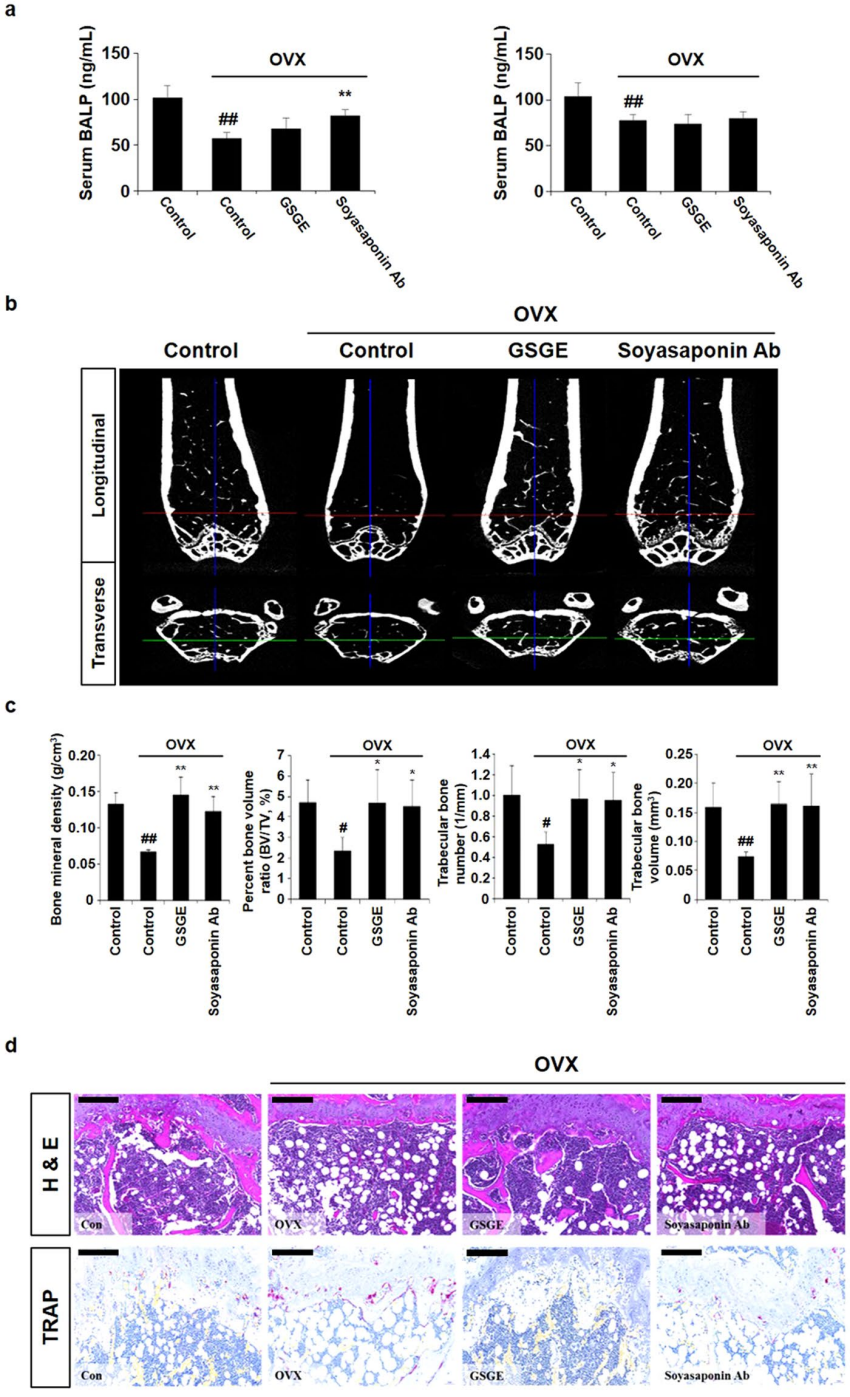
Soyasaponin Ab improves OVX-mediated bone loss. (**a**) Serum BALP and 17β-estradiol concentration was estimated using an ELISA kit ^##^*p* < 0.01 (versus Con group); **p < 0.01 (versus OVX group). (**b**) Longitudinal and transverse µ-CT images of Con, OVX, GSGE, and Soyasaponin Ab from left to right, and (**c**) bone morphometric parameters for µ-CT analysis assessed using 3D images analyzer. ^#^*p* < 0.05; ^##^*p* < 0.01 (versus Con group); *p < 0.05; **p < 0.01 (versus OVX group). (**d**) Mice were euthanized 10 weeks after GSGE and soyasaponin Ab treatment, and femurs were dissected, fixed, decalcified, and sectioned. Sections were stained with H&E. Stained bone sections were photographed under a light microscope (upper panel; scale bar, 200 μm) and for TRAP activity (bottom panel; scale bar, 200 μm).

The bone-protecting effect of soyasaponin Ab was evaluated using an ovariectomized mouse model. Serum BALP level increased in GSGE and soyasaponin Ab groups (Fig. 6a; left graph), although no significant change in serum 17β-estradiol level was seen among the experimental groups (Fig. 6a; right graph). Reconstructed three-dimensional images of distal femurs showed that the trabecular pattern of GSGE- and soyasaponin Ab-treated groups was improved bone density compared to that of the OVX group (Fig. 6b). Bone morphometric parameters used in µ-CT analysis of GSGE and Soyasaponin Ab groups increased to the levels in the Con group (Fig. 6c). Histological assessments revealed that OVX decreased the trabecular bone dimension, and osteoporosis progressed to the growth plate region. In contrast, GSGE and soyasaponin Ab treatment increased the relative trabecular bone dimension and suppressed osteoclast differentiation compared to an OVX group (Fig. 6d). These results indicate that soyasaponin Ab plays an important role in bone formation as a major component of GSGE.

## Discussion

The incidence of senile osteoporotic fracture can be reduced by prescribing anabolic agents such as parathyroid hormone. However, the use of anabolic agents is limited by cost, subcutaneous injection, and concerns about long term safety^10^. Complementary and alternative medicines for reducing these limitations might be found in natural products with potential anabolic effects. Studies have attempted to identify natural pharmacological compounds or nutritional substances for prevention and treatment of osteoporosis with minimal adverse effects, but the effects of such materials on skeletal fragility are weak^11^. Strategies for enhancing the efficacy of natural products could be promising therapeutic tools for reducing the risk of bone metabolic disorder, such as osteoporosis.

Soy is widely used in healthy foods and nutritional support. Soy contains high protein and oil contents and bioactive components known as phytochemicals. Several studies have shown that SE, SGE, and their bioactive ingredients cause improvement of cardiovascular disease, cancer, glucose tolerance, and bone health and have antioxidant activities^12,13^. Germination of soy germ is reported to increase the levels of bioactive and nutritional components^14^. Although previous studies have shown that phytochemicals such as isoflavones and soyasaponin prevent bone loss through anabolic activation of osteoblasts and antiresorptive action against osteoclasts^13,15^, the detailed mechanism and *in vivo* evidence of GSGE and its bioactive ingredient are still poorly understood. This study reported on the mode of action in osteogenesis and the restoration of bone loss in postmenopausal osteoporosis through treatment with GSGE and soyasaponin Ab.

The differentiation of osteoblasts from mesenchymal stem cells or osteoprogenitor cells in bone marrow is mainly regulated by BMP-2, a transforming growth factor-β (TGF-β) originally described as a bone morphogens, that enhances ALP activity in a pluripotent mesenchymal precursor cell line, C2C12^16^. BMP-2 triggers osteoblast differentiation, which is considered to be a major target for healing bone loss. In our study, GSGE synergistically improved BMP-2-induced osteoblast differentiation of C2C12 cells in a dose-dependent manner compared with anabolic effects of SE or SGE.

BMP-2 stimulation during osteoblast differentiation is highly specific to osteoblastic transcription factors such as Runx2 and Osx. These transcription factors are pivotal in the regulation of molecules such as ALP and OCL in osteoblast differentiation^17,18^. The critical function of Runx2 in osteoblast formation has been demonstrated in knockout mice and those with ectopic expression of Runx2 and by studying Runx2 mutations in humans^19,20^. Runx2 haploinsufficiency is associated with cleidocranial dysplasia in humans^19^. Runx2 heterozygous knockout mice have osteopenia as adults due to low bone turnover caused by diminished osteoblastic function^21^. Conversely, ectopic expression of Runx2 leads to endochondral ossification in parts of the skeleton^22^. Another major transcription factor, Osx, is essential for differentiation of preosteoblasts into fully functioning osteoblasts Formation of cortical and trabecular bone is abolished by absence of Osx^23^. Therefore, Runx2 and Osx are essential factors for osteoblast differentiation and mature bone formation. In our study, the two transcription factors Runx2 and Osx showed synergistically enhanced induction of mRNA and protein by GSGE treatment in the presence of BMP-2 during osteoblast differentiation. The stimulatory effect of GSGE by induction of Runx2 and Osx was confirmed by evaluating the transcription of ALP and OCL osteoblast-specific markers. The results showed that acceleration of osteoblast differentiation by GSGE involved induction of Runx2 and Osx, which are major transcription factors.

BMP-2 transmits osteogenic signaling through a Smad-dependent pathway^24^. Smads are major signal transducers for the serine/threonine kinase receptor. Phosphorylation and translocation of Smad by BMP-2 induce BMP-mediated signaling for bone formation, resulting in osteoblastogenesis-related gene expression^25^. Furthermore, BMP-activated Smad interacts with Runx2 and further induces osteoblast differentiation^25^. In this study, GSGE induced BMP-stimulated phosphorylation of Smad. These results demonstrate that the anabolic effect of GSGE on osteoblast differentiation could result from its ability to stimulate the Smad-Runx2/Osx signaling axis in bone formation.

Soyasaponins are mainly classified into groups A and B. Soyasaponins in group A are glycosylated at the carbon-3 and carbon-22 positions of soyasapogenol A, whereas 2,3-dihydro-2,5-dihydroxy-6-methyl-4H-pyran-4-one-conjugated group B soyasaponins are glycosylated at the carbon-3 position^26^. Isomers of each group depend on the number of glycosides attached to soyasapogenol A and acetyl groups attached to the glycosides^26^. Our study found that GSGE had a higher concentration of soyasaponin Ab than SE and SGE. The biological activity in osteoblast differentiation of soyasaponin Ab in GSGE was assessed. The physiological functions of soyasaponins are reported to be anti-oxidative with anti-kidney-disease progression, anti-inflammatory, and anti-tumor effects and to result in reduced blood glucose level, renin inhibition, and hepatoprotection^27–29^. Although efficacy studies of soyasaponin on improvement of osteoporosis have been reported, the detailed anabolic effects and possible molecular mechanisms of soyasaponin Ab have not been explored. For bone formation effects of GSGE, soyasaponin Ab enhanced BMP-2-induced osteoblast differentiation and expression of Runx2, Osx, ALP, and OCL in a dose-dependent manner. These results indicate that the high soyasaponin Ab content in GSGE could be a bioactive ingredient for bone formation during osteoblast differentiation.

The *in vitro* results of GSGE and soyasaponin Ab prompted us to investigate their *in vivo* efficacy and possible effects on postmenopausal osteoporosis. OVX-induced bone loss in mice is widely used as a model of postmenopausal osteoporosis and is validated as a clinically relevant model of this condition in humans. For OVX-stimulated bone loss in the animal model used in this study, GSGE and soyasaponin Ab significantly protected mice against OVX-induced bone loss. Kim *et al*.^14^ reported that germinated soy germ has highly increased bioactive and nutritional components such as isoflavones, soyasaponins, and free amino acids. Previous studies have shown that isoflavones stimulate osteoblast proliferation and activation and suppress osteoclast formation and activation^13,30^. For these reasons, the bone-protecting effect of soyasaponin Ab was weaker than that of GSGE administration, although soyasaponin Ab was excellent in the bone-protecting effect. In addition, the intake of GSGE and soyasaponin Ab improved bone morphometric parameters including bone mineral density, percent bone volume ratio, trabecular bone number, trabecular bone volume, and trabecular bone thickness. Histopathological evaluation and serum biomarker assays revealed that GSGE and soyasaponin Ab had *in vivo* bone-forming effects with no changes in serum 17β-estradiol level. Considering the adverse effects caused by hormone replacement therapy, which involves the administration of synthetic estrogen, this *in vivo* result is especially worth noting. These results demonstrated that GSGE and soyasaponin Ab have a high possibility of preventing and improving postmenopausal osteoporosis while minimizing the burden of adverse effects that can be caused by hormone replacement therapy.

In summary, this is the first study to show that GSGE and soyasaponin Ab might be relevant for osteoblast-mediated bone loss diseases because of the anabolic effect of GSGE and its bioactive component, soyasaponin Ab. In particular, the bone-forming effect of GSGE was associated with induction of Smad-Runx2/Osx signaling molecules required for osteoblast differentiation. Moreover, GSGE and soyasaponin Ab showed *in vivo* bone protection effects in an OVX-induced bone-loss animal model. The bone-protecting effects of GSGE were considered to be contributed mainly by soyasaponin Ab, although additional studies are required to substantiate the functional components in GSGE for osteoblastogenic activity. Unlike hormone replacement therapy, no adverse effects were expected since GSGE is a substance extracted from plants. The results presented here suggest that GSGE and soyasaponin Ab could be promising therapeutic substances for preventing and improving bone loss disorders including postmenopausal osteoporosis.

## Materials and Methods

### Preparation of germinated soy germ extracts

Separation and germination of soy germ were conducted as described by Kim *et al*.^14^. The Daepung soybean variety was dried at 35 °C in an oven for 72 hours to a moisture content of approximately 7.4–10.2%. Soybeans were processed with a soybean crusher (Dae-Ruck Food Machine, Daegu, Korea) specifically designed to crack whole soybeans, producing about five to six particles of cotyledon and removing the seed coat. After cracking, soybean mixtures were passed through 2.36, 2.0, and 1.18 mm sieves to separate the soy germ. Germ was washed thoroughly and poured into a 40 × 50-cm net sack and germinated at room temperature (25 ± 1 °C) for 24 hours under running water. Germinated soy germ (GSG) was collected and freeze-dried, ground in a laboratory test mill (Brabender, Germany), and passed through a 100-mesh flour sieve. Preparation of GSGE was as described by Kim *et al*.^14^. GSG flour was defatted with hexane for 48 hours at room temperature, and 1 kg was twice extracted with methanol (2 L) using a reflux apparatus at 90 °C for 6 hours. Extracts contained a large amount of highly soluble substances as well as isoflavones and soyasaponins. GSGE was stored in a fume hood at room temperature for 24 hours to remove such materials, inducing condensation and precipitation of proteins and sugars. GSGE was filtered with cotton wool and filter paper (Whatman No. 3) to remove proteins and saccharides such as sugars. Filtered extracts were centrifuged at 3000 rpm for 20 minutes and concentrated in a rotary evaporator.

### Reagents and antibodies

Recombinant human bone morphogenetic protein-2 (rhBMP-2) and ALP antibody were purchased from R&D Systems (Minneapolis, MN). Penicillin, streptomycin, cell culture medium, and fetal bovine serum (FBS) were purchased from Invitrogen Life Technologies (Carlsbad, CA). Antibodies against actin, Smad, and secondary antibody conjugated to horseradish peroxidase were purchased from Santa Cruz Biotechnology (Dallas, TX). Osx antibody was obtained from Abcam (Cambridge, UK). All other antibodies were from Cell Signaling Technology (Beverly, MA).

### Cell culture and osteoblast differentiation

All experiments were as described previously with modifications^31^. Mouse mesenchymal precursor C2C12 cells were from the American Type Culture Collection (Manassas, VA). C2C12 cells were maintained in alpha minimum essential medium (α-MEM) with 10% FBS, 100 U/mL penicillin, and 100 μg/mL streptomycin. Cells were seeded in 96-well plates at 2.5 × 10^3^ cells/well or in 6-well plates at 2.5 × 10^5^ cells/well. After 1 day, cells were differentiated by replacing the medium with α-MEM containing 5% FBS and rhBMP-2 (100 ng/mL) with soy extracts (SE), soy germ extracts (SGE), or GSGE at the indicated dose. Osteoblastic bone formation was observed by ALP staining.

### Alkaline phosphatase staining and activity assays

ALP is an early biomarker of osteoblast differentiation. After differentiation for 3 days, cells were washed twice with phosphate-buffered saline (PBS), fixed with 10% formalin in PBS for 5 minutes, rinsed with deionized water, and stained using an ALP Kit (Sigma-Aldrich). To measure ALP activity, differentiated cells were washed twice with PBS, fixed with 10% formalin in PBS for 5 minutes, rinsed with PBS, and measured using a one-step PNPP substrate solution (Thermo Scientific, MA) according to the manufacturer’s protocol.

### Cell viability assays

C2C12 cells were seeded in 96-well plates at 2.5 × 10^3^ cells/well, in triplicate. After treatment with the indicated concentrations of SE, SGE, GSGE, and soyasaponin Ab, cells were incubated for 3 days, and cell viability measured using a Cell Counting Kit 8 (CCK-8) according to the manufacturer’s protocol. CCK-8 assay kits were purchased from Dojindo Molecular Technologies (Rockville, MD).

### RNA isolation and real-time polymerase chain reaction analysis

Real-time polymerase chain reaction (PCR) was performed as described previously^32^. Primers were chosen using the online Primer3 design program. Primer sets used in this study are shown in Supplementary Table S1. Total RNA was isolated with TRIzol reagent, and first-strand cDNA was synthesized with a RevertAid First Strand cDNA Synthesis Kit (Thermo Scientific) according to the manufacturer’s recommended protocol. SYBR green-based quantitative PCR (qPCR) was performed using the Thermo Scientific Quantstudio^®^ 5 Real-Time PCR Detection System and Applied Biosystems Power-up SYBR green PCR master mix (Thermo Scientific). All reactions were performed in triplicate, and data were analyzed using the 2^−ΔΔCT^ method. Hypoxanthine phosphoribosyltransferase 1 (HPRT1) and glyceraldehyde 3-phosphate dehydrogenase (GAPDH) were used as internal standard genes. Statistical significance was determined using Student’s *t*-test with HPRT1/GAPDH-normalized 2^−ΔΔCT^ values; differences were considered significant at *p* < 0.05.

### Western blot analysis

Western blots were as described previously^32^. Briefly, the cultured cells were washed with ice-cold PBS and lysed in lysis buffer (50 mM Tris-HCl, 150 mM NaCl, 5 mM EDTA, 1% Triton X-100, 1 mM sodium fluoride, 1 mM sodium vanadate, and 1% deoxycholate) with protease inhibitors. After centrifugation at 15,000 × *g* for 15 minutes, protein quantification of supernatants was performed using a detergent-compatible protein assay kit (Bio-Rad, Hercules, CA). Proteins were denatured, separated by sodium dodecyl sulphate-polyacrylamide gel electrophoresis, and transferred to a polyvinylidene difluoride membrane (Merck Millipore, Darmstadt, Germany). After incubation with antibody, membranes were developed using SuperSignal West Femto Maximum Sensitivity Substrate (Thermo Scientific) and visualized with an LAS-4000 luminescent image analyzer (GE Healthcare Life Sciences, Little Chalfont, UK). Actin and GAPDH were used as loading controls.

### UHPLC-CAD analysis

SE, SGE, and GSGE were dissolved in methanol to 1 mg/mL and filtered through 0.2-μm filter units before ultra-high performance liquid chromatography (UHPLC)-changed aerosol detection (CAD) analysis. Soyasaponin Ab analysis was conducted using a reverse-phase UHPLC (Dionex Ultimate 3000, Thermo Scientific) equipped with a Acclaim^TM^ RSLC Polar Advantage II (2.2 μm 120 Å 2.1 × 150 mm) column. The mobile phase was 0.1% acetic acid in water (A) or 0.1% acetic acid in acetonitrile (B). The solvent flow rate was 0.7 mL/min, and the column temperature was set at 35 °C. The gradient was programmed as: 0–2 min, 10% B; 5 min, 20% B; 15 min, 30% B; 20 min, 30% B; 40 min, 70% B; 50 min, 100% B; 50.1 min, 10% B, held for 9.9 min before returning to the initial conditions. Following injection of 2 μL of sample, eluted soyasaponin Ab was detected using a charged aerosol detector (CAD, Corona Veo, Thermo Scientific). The standard of soyasaponin Ab was purchased from ChemFaces (Wuhan, China). The calibration curve was plotted by peak area versus concentration of soyasponin Ab. To prepare the standard solution, soyasaponin (5, 10, 20, 40, 80 mg/mL) were dissolved in MeOH. The linear regression equations were calculated with *y* = *ax* ± *b*, where *x* was concentration and *y* was the peak areas of soyasaponin Ab. The linearity was established by the coefficient of determination (*R*^2^).

### Animal modeling study design

All animal experiments were performed in accordance with the relevant ethical guidelines set by the Institutional Animal Care and Use Committee (IACUC) of Konkuk University. All experimental methods were approved by the IACUC of Konkuk University (KU15103). Female 8-week-old C57BL/6 mice (n = 72) weighing an average of 25 g were purchased from Orient Bio (Seongnam, Korea). Ovariectomy (OVX) or sham operation was performed at the age of 9 weeks. OVX was conducted after anesthetization with xylazine HCl and isoflurane (Byer Korea, Kyungkido, Korea). Sham-operated animals had their ovaries exposed but not removed. The OVX or sham-operated mice were randomized and divided into experimental groups (n = 8 per group). For GSGE treatments, experimental groups were Con (sham-operated), OVX (only OVX, untreated), 0.1 mg (OVX + GSGE 0.1 mg/kg in saline), 1 mg (OVX + GSGE 1 mg/kg in saline), and 5 mg (OVX + GSGE 5 mg/kg in saline). Treatment was initiated 1 week after the operation and continued for 12 weeks. For soyasaponin Ab treatment, the experimental groups were Con (sham-operated), OVX (only OVX, untreated), GSGE (OVX + GSGE 5 mg/kg in saline), and Soyasaponin Ab (OVX + soyasaponin Ab 5 mg/kg in saline). Treatment was initiated 1 week after the operation and continued for 10 weeks. Experimental groups received injections of solutions at the indicated dosages for 6 days per week. Abnormal clinical signs and general behavioral changes were observed once a day, and body weight was measured on the day of dosing and every week during the experimental period.

### Microcomputed tomography analysis

Femurs from all experimental group members were examined by microcomputed tomography (µ-CT). Examinations were used to determine changes in trabecular bone and were carried out using a SkyScan1173 (SKYSCAN, Belgium) at 8-µm resolution. The degree of bone density based on µ-CT was quantified using mean gray value and standard deviation of the region of interest. Morphometric parameters were bone mineral density (g/cm^3^), percent bone volume ratio (bone volume/tissue volume, %), trabecular bone number (1/mm), trabecular bone volume (mm^3^), and trabecular bone thickness (mm), obtained using On-demand software (CyberMed Inc., Korea).

### Histopathology and serum biochemistry

All animals were anesthetized with ether and euthanized 10 or 12 weeks after treatment. Blood was collected from abdominal veins through laparotomy incision, and right femurs were collected for examination. Serum and bone samples were stored at −70 °C. Specimens were prepared for histopathological observation. Right femurs were fixed in 10% neutral buffered formalin and soaked in decalcifying solution and buffered formic acid before being subjected to routine tissue processing. Specimens were cut into 4-µm sections and stained with hematoxylin and eosin. Trabecular bone dimensions in histopathological profiles of diaphyseal regions were calculated using the Visus image analysis program (Visus, Foresthill, CA). Specimens were stained with tartrate-resistant acid phosphatase (TRAP) staining solution and counterstained with hematoxylin in order to determine the anti-osteoclastic differentiation effects of GSGE and soyasaponin Ab. Osteoclast number was quantitated by counting TRAP-positive multinucleated cells, defined as osteoclasts in this study. The concentration of serum bone-specific alkaline phosphatase (BALP) and serum 17β-estradiol was measured with an enzyme-linked immunosorbent assay (ELISA) using Mouse Bone-specific alkaline phosphatase ELISA kit (CUSABIO, Wuhan huamei biotech, China) and 17β-estradiol ELISA kit (Abcam), respectively.

### Statistical analysis

Quantitative values are mean ± standard deviation. Each experiment in triplicate was performed three to five times. Several figures show results from one representative experiment. Statistical differences were analyzed using Student’s *t-*test, and a value of *p* < 0.05 was considered significant.

## Electronic supplementary material

Supplementary Information
