# Supplementary material for: Germinated soy germ with increased soyasaponin Ab improves BMP-2-induced bone formation and protects against in vivo bone loss in osteoporosis
Source: Sci Rep. 2018 Aug 28;8:12970. doi: 10.1038/s41598-018-31118-w (PMC6113227; doi:10.1038/s41598-018-31118-w)
Supplement: Supplementary file 1 — Supplementary Information [file 41598_2018_31118_MOESM1_ESM.pdf]

Supplementary Information

Germinated soy germ with increased soyasaponin Ab improves BMP-2-induced bone formation and protects against *in vivo* bone loss in osteoporosis

Chan-Woong Choi[1,+], Sik-Won Choi[2,+], Han-Jun Kim[1], Kwnag-Sik Lee[2,3], Shin-Hye Kim[2,4], Sun-Lim Kim[5], Sun Hee Do[1,*], Woo-Duck Seo [2,*]

[1]College of Veterinary Medicine, Konkuk University, Seoul 05029, Republic of Korea; [2]National Institute of Crop Science, Rural Development Administration, Wanju-Gun 55365, Republic of Korea; [3]College of Crop Science and Biotechnology, Dankook University, Cheonan 31116, Republic of Korea; [4]Department of Biological Sciences, College of Natural Science, Chonbuk National University, Jeonbuk 55000, Republic of Korea; [5]Dapartment of Central Area Crop Science, National Institute of Crop Science, Rural Development Administration, Suwon 16429, Republic of Korea

[+]CWC and SWC contributed equally to this study.

***Co-corresponding author:**

Sun Hee Do, DVM, PhD

Department of Clinical Pathology, College of Veterinary Medicine, Konkuk University, Seoul 05029, Republic of Korea

Tel: +82-2-450-3706

Fax: +82-2-452-3706
E-mail address: shdo@konkuk.ac.kr

Woo-Duck Seo, PhD
Laboratory of Crop Resource Development, Division of Crop Foundation, National Institute of Crop Science, Rural Development Administration, Jeonbuk 55365, Republic of Korea
Tel: +82-63-238-5333
Fax: +82-63-238-5335
E-mail: swd2002@korea.kr

Supplementary Table 1. Primers used in this study

Target Gene	Forward Primer (5′–3′)	Reverse Primer (5′–3′)
Runx2	GACTGTGGTTACCGTCATGGC	ACTTGGTTTTTCATAACAGCGGA
Osx	CTTCCACTTCGCCTGCACCC	GGAGCATAGGAACTAGGCAC
ALP	GATGGCGTATGCCTCCTGCA	CGGTGGTGGGCCACAAAAGG
OCL	AGGGAAACCTCATCCGTTTG	GAGCCGGAAATAAGGCACAG
GAPDH	ACCACAGTCCATGCCATCAC	TCCACCACCCTGTTGCTGTA
HPRT1	TGCTCGAGATGTCATGAAGG	AGAGGTCCTTTTCACCAGCA

Supplementary Table 2. Recovery study of soyasaponin Ab from GSGE

Compound	Concentration in sample (mg)	Standard added (mg)	Recovery (mg)		Recovery (%)	RSD[b] (%)
			Expected	Actual[a]		
Soyasaponin Ab	230.4±7.4	5.0	235.4	232.8±7.9	98.9	0.8
	230.4±7.4	25.0	255.4	251.4±8.5	98.4	1.1
	230.4±7.4	50.0	280.4	277.2±9.4	98.9	0.7

[a] values are means ± standard deviation of triplicate samples , [b] Relative standard deviation

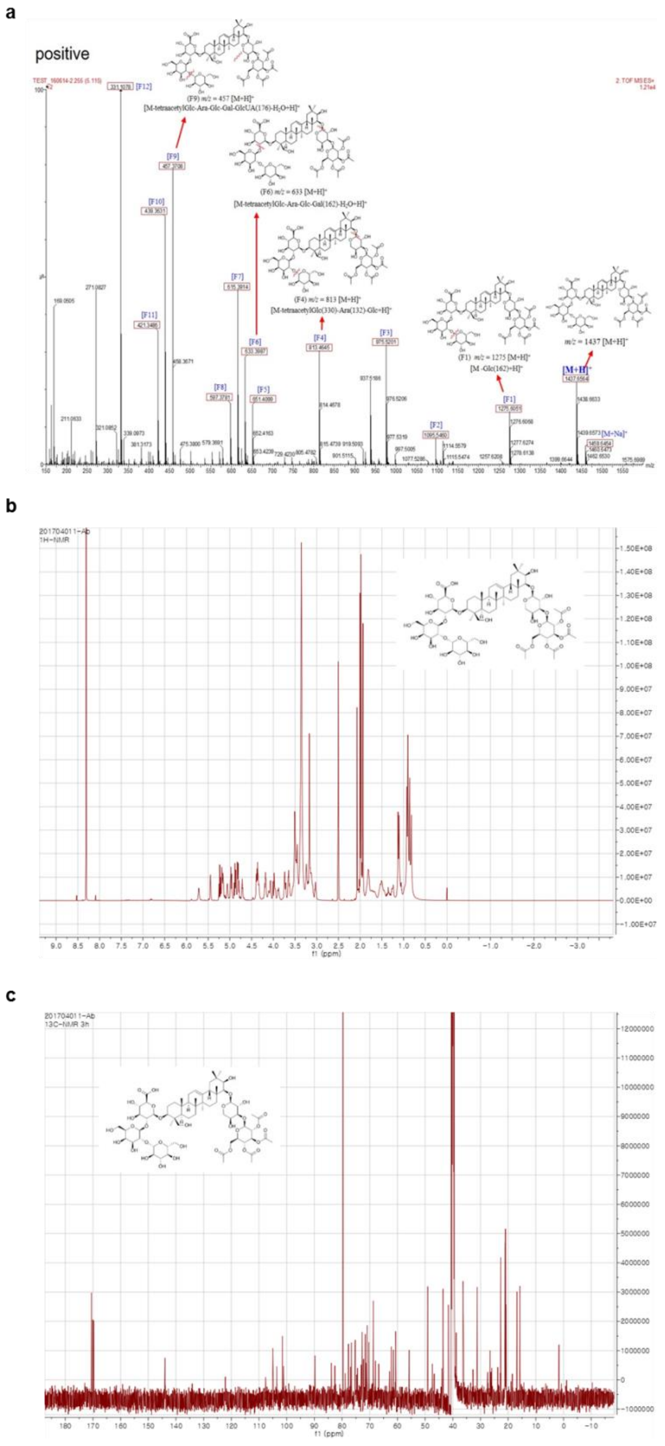

Supplementary Figure 1. Identification and characterization of soyasaponin Ab. UPLC-QTOF-MS/MS (a), ^{1}H-NMR (b), and ^{13}C-NMR (c) spectra of soyasaponin Ab.
